# Evaluation of the Textural Parameters of Zeolite Beta in LDPE Catalytic Degradation: Thermogravimetric Analysis Coupled with FTIR Operando Studies

**DOI:** 10.3390/molecules25040926

**Published:** 2020-02-19

**Authors:** Kamila Pyra, Karolina A. Tarach, Ewa Janiszewska, Dorota Majda, Kinga Góra-Marek

**Affiliations:** 1 Faculty of Chemistry, Jagiellonian University, 30-387 Kraków, Poland; karolina.tarach@uj.edu.pl (K.A.T.); majda@chemia.uj.edu.pl (D.M.); 2 Faculty of Chemistry, Adam Mickiewicz University, 61-614 Poznan, Poland

**Keywords:** hierarchical zeolites, FTIR operando spectroscopy, polyethylene cracking

## Abstract

Zeolite-based catalysts are globally employed in many industrial processes, such as crude-oil refining and bulk chemical production. In this work, the cracking of low-density polyethylene (LDPE) was thoroughly followed in a FTIR operando study to examine the catalytic efficiency of purely microporous zeolites β of various textural characteristics. To provide complementary and valuable information on the catalytic activity of the zeolite β studied, the thermogravimetric analysis results were compared with yields of the products generated under operating conditions. The reaction products were analyzed via GC–MS to determine the hydrocarbon chain distribution in terms of paraffin, olefins, and aromatics. The individual impact of textural and acidic parameters on catalytic parameters was assessed. The accumulation of bridging hydroxyls of high strength in the zeolite β benefited the decrease in polymer decomposition temperature. Through a strategic comparison of purely microporous zeolites, we showed that the catalytic cracking of LDPE is dominated by the acidic feature inherent to the microporous environment.

## 1. Introduction

The acidic characteristics of zeolite β originate from its broad range of Si/Al ratios and its particular structure disordered in one dimension, as a result of polymorph intergrowth [1,2,3,4]. The three-dimensional interconnected channel system built up from 12 rings classifies zeolite β as a large-pore three-dimensional (3D) zeolite. Zeolite β is widely reported as a solid acid catalyst, particularly addressed to cracking [5,6,7], (hydro)isomerization of alkanes [8,9], and alkylation of aromatics [10,11,12]. Aguado et al. [13] examined products gained from catalytic cracking of low- and high-density polyethylene (LDPE and HDPE) and polypropylene (PP) over zeolites β synthesized via various methods, evidencing good selectivity toward C_5_–C_12_ hydrocarbons. Manos et al. [14] using zeolites Hβ, HZSM-5, and USY for the catalytic degradation of HDPE showed that the product distribution depends on the zeolite structure. In addition, they indicated the high selectivity of catalyst β to the C_5_–C_12_ alkanes.

Understanding the structure–activity relationship of zeolitic catalysts in many reactions still remains an engaging research topic for the scientific community. The activity of acidic catalysts depends not only on the specific structural and topological properties but also on the nature of active sites. The concentration, accessibility, and acid strength of the Brønsted and Lewis sites are usually identified in the spectroscopic or thermogravimetric adsorption studies of basic probe molecules. The individual effects of textural and acidic features on the catalytic performance can be assessed only if the respective impacts are decoupled [15]. Therefore, the straightforward correlations between catalytic activity and properties of zeolites can be determined within the same zeolitic topology.

Through a strategic comparison of zeolites β with tuned microporous characteristics, we showed that the catalytic cracking of LDPE over 12-ring zeolite β is ruled by the acidic feature. Different synthesis methods were applied to obtain zeolites β possessing tuned microporous features [16,17,18]. The zeolites were synthesized through the protozeolytic unit-based method. Then, the organosilane functionalization was done to modify the morphological and textural zeolite features and prevent crystal growth during further crystallization. To follow the influence of functionalization, the zeolite without this step was also synthesized. Finally, the acidic treatment over the functionalized sample was done to adjust the acidic properties. Commercially available zeolite served as the reference material. This work aims to show that the attenuation of the dispersive interactions between the fragments of LDPE chains and the Si(OH)Al in the confined voids of micropores significantly affects the catalytic behavior.

## 2. Results

### 2.1. Physicochemical Properties of the Catalysts

**Figure 1:**
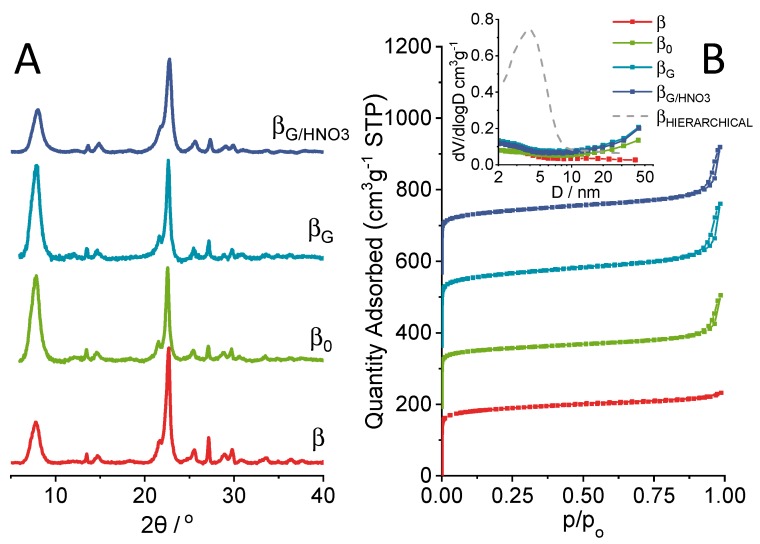
XRD patterns (**A**) and N_2_ adsorption–desorption isotherms with pore size distribution (inset) (**B**) of the studied samples (β_HIERARCHICAL_ sample added for comparison [5]).

Well-resolved reflections representative of the BEA^*^ structure (Figure 1A) were found in the XRD patterns of the commercial zeolite β and zeolites obtained from the protozeolytic unit-based synthesis method (β_0,_ β_G_). All the zeolites exhibited both type I and type IV N_2_-isotherms corresponding to the porosity originating from the highly developed microporous characteristics (V_micro_ and S_micro_, Table 1, Figure 1B). The high-pressure hysteresis loop pointed to the presence of interparticle porosity [19]. The mild treatment of β_G_ with HNO_3_ solution slightly improved the microporous parameters (V_micro_ and S_micro_), while the value of Si/Al ratio was kept within the range of 19–22 for all the zeolites studied. The studied zeolites were purely microporous materials, and neither functionalization with organosilane nor acid treatment resulted in the formation of mesopores, as can be seen in pore size distribution (PSD) chart (Figure 1B, inset). For comparison purposes, the PSD for hierarchical zeolite β obtained by caustic treatment is also presented [5].

**Figure 2:**
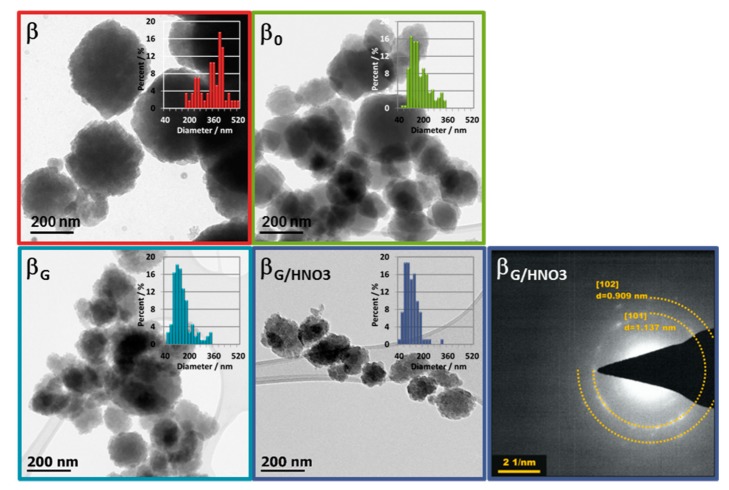
TEM micrographs of all studied samples and diffraction pattern of β_G/HNO3_ zeolite taken from the selected area.

The commercial zeolite β consisted of particles with a size of 200–400 nm (Figure 2). The β_0_ zeolite prepared using the protozeolytic unit-based method possessed smaller crystals (100–200 nm), which undoubtedly indicates that the presence of more nucleation centers during synthesis leads to smaller particles of zeolite. Functionalization of protozeolytic units by an organosilane significantly changed the morphology of β_G_ sample. The agglomerated globular structures of 100–150 nm possessed a more uniform distribution of size. The small crystals covered those globular structures, as easily distinguishable in the STEM micrographs (Figure S1, Supplementary Materials). The acid treatment affected the particles size distribution by increasing the fraction of the particles with a size below 100 nm, which slightly increased the external surface area and volume values (S_EXT_, V_EXT_). The acid treatment of β_G_ also resulted in Al abundance differentiation in the β_G/HNO3_ sample. The transmission electron microscopy-based energy-dispersive X-ray (EDX) analysis of single zeolite crystals and ICP-OES elemental analysis showed the lowered content of Al (0.84 wt.%) with respect to non-leached zeolite β_G_ (1.25 wt.%). Further distinction between changes in concentration of differently coordinated aluminum species was derived from pyridine and low-temperature CO adsorption followed by FTIR spectroscopy (Section 2.2).

### 2.2. Acidic Properties: Pyridine and Carbon Monoxide Sorption Studies

**Figure 3:**
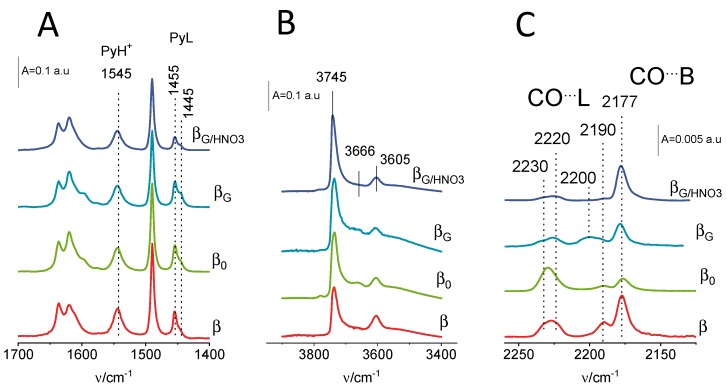
IR spectra of zeolites studied collected (**A**) during quantitative Py sorption studies, (**B**) in the region of O–H stretching vibrations. and (**C**) during the saturation of Lewis acid sites with carbon monoxide.

In addition to specific sorption properties derived from the size and spatial arrangement of pores, the applicability of the zeolites is also ruled by their acidic properties. The acidic property is decisive especially in cracking reactions. Several methods were developed to follow the origin and concentration of both Brønsted and Lewis acid sites. Among them, FTIR spectroscopy, involving the use of probe molecules, is of the highest significance. Pyridine (Py) [21] is a suitable probe molecule for the quantitative analysis of surface acidity of medium and wide pore zeolites. The assessment of textural and acidic parameters influences, in an individual manner, the catalytic performance required to decouple the respective impacts. Pyridine adsorption FTIR experiments (Table 2) assessed, in qualitative and quantitative terms, the protonic (the 1545-cm^−1^ band) and Lewis (1455–1445-cm^−1^ bands) acidity (Figure 3A). The studied zeolites accommodated a comparable number of protonic sites. The total concentrations of acid sites (C_B_ + C_L_) determined by Py in all the zeolites matched well with the values of the Al content obtained from the chemical analysis (Table 2). The variations between the Al content and the acid site concentration did not exceed 15%; thus, each Al atom was supposed to form either an acidic Si(OH)Al group or a Lewis acid site, both easily accessible to the Py molecule. Certain deviations in the number of the sites quantified by Py and Al atoms calculated from the zeolite composition may have originated from the presence of non-acidic Al-species detected in OH-groups FTIR spectra as the band at 3666 cm^−1^, albeit with negligible intensity (Figure 3B). It should be noted that the relatively high concentration of Lewis acid sites in all the zeolites β resulted from the partial dealumination occurring during removal of the high amounts of SDAs (structure-directing agents). Treatment of zeolite β_G_ with an acidic solution led to the partial removal of Al species serving as Lewis acid sites; however, no perturbation in the number of protonic sites was observed.

The acid strength of studied zeolites was determined based on Py thermo-desorption and low-temperature CO adsorption experiments followed by FTIR spectroscopy. The Py_450_/Py_170_ values (Py_450,_ Py_170_ represent the PyH^+^ ion band intensities upon the desorption at the temperatures indicated) (Table 2) clearly point out that the strength of protonic sites in all the protozeolytic unit-synthesized zeolites was higher than in the commercial material β. Additionally, carbon monoxide was applied to probe the strength of the Si(OH)Al acid sites. The significantly lower value of the proton affinity (PA) of carbon monoxide (CO, PA = 141.9 kcal∙mol^−1^) than Py pyridine (Py, PA = 204 kcal∙mol^−1^) [22,23] resulted in the interaction of CO with the Si(OH)Al groups via a weak H-bond, thus leading to the formation of the Si(OH^•••^CO)Al adducts. Saturation of all protonic sites with carbon monoxide at −130 °C resulted in the downshift of the IR band of the acidic Si(OH)Al hydroxyls due to their hydrogen bonding to CO molecules; therefore, the ∆υ(_CO•••OH_) can be taken as a measure of the acid strength. The commercial zeolite β possessed the Brønsted sites of the lowest strength as documented by the lowest value of ∆υ(_CO•••OH_). The highest values ∆υ(_CO•••OH_) were observed for the zeolite β_G/HNO3_, i.e., the material with the highest value of micropore volume. The extraction of aluminum extra-framework material via acidic solution treatment resulted in a further upsurge of the acid strength of Si(OH)Al hydroxyls (Py_450_/Py_170_ and ∆υ(_CO•••OH_), Table 2)_._ The contribution of the dispersion interaction between the small CO molecules and zeolite wall for the Si(OH^•••^CO)Al adsorption complexes can be assumed equal for all β zeolites studied. As a result, the ∆υ(_CO•••OH_) values were independent of the dispersion contributions, and they strictly represent the trends in the acid strength alteration.

The differences in the speciation of Lewis acid sites was demonstrated by the presence of two PyL bands at 1445 and 1455 cm^−1^. From the weak intensity of the 1445-cm^−1^ band, we can conclude the presence of a small population of the Lewis sites of low strength. The additional insight into the Lewis acid site nature was provided in the CO adsorption experiments, in which small doses were adsorbed up to the total saturation of all the Lewis acid sites, i.e., the maximum intensities of the bands in the 2250–2180-cm^−1^ frequency region and the appearance of the 2177-cm^−1^ band of the CO interacting with Brønsted acid sites (Figure 3C). Numerous studies on zeolites β focused on the role of the octahedrally coordinated aluminum species, distinguishing them as extra-framework- or framework-associated, depending on the aluminum’s ability to reversibly change its coordination. The framework-associated octahedrally coordinated aluminum in protonic zeolites is created by partial hydrolysis of framework Si–O–Al bonds able to quantitatively revert to tetrahedral coordination when heating or ion-exchanging the zeolite to Na^+^ or NH_4_^+^ form [24,25,26,27,28]. The octahedrally coordinated aluminum species are believed to be present only in the case of proton-exchanged zeolites. Two close Brønsted acid sites are, therefore, needed to generate the defective site able to change the aluminum coordination into octahedral [29]. The Lewis acid sites in all the zeolites studied mostly originated from the dehydroxylation process (represented by the CO bands at 2230 and 2220 cm^−1^) and can be, therefore, considered as framework-associated three-fold coordinated aluminum atoms. The Al species represented by the CO bands at 2200–2190 cm^−1^ significantly populated only in β_G_ were attributed to extra-framework- and/or framework-associated octahedral species. This attribution is in good agreement with the observation that solely the latter species, as well as Al-OH species (represented by 3666-cm^−1^ bands, Figure 3B), turned out to be easily leached by acid treatment.

### 2.3. Catalytic Performance: Thermogravimetric and Operando IR Studies

**Figure 4:**
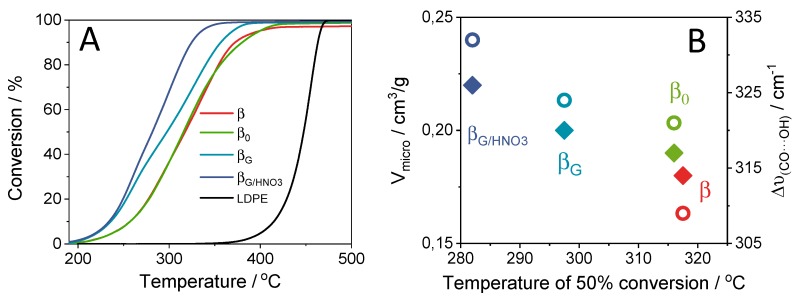
(**A**) Conversion curves of low-density polyethylene (LDPE) cracking on studied zeolites. The thermal cracking of LDPE is included for comparison. (**B**) The correlation between catalytic activity in polyethylene decomposition (temperature for 50% conversion) and the strength of sites (open symbols) and V_micro_ (solid symbols).

The results of the LDPE catalytic cracking over zeolites studied are given in Figure 4, where the LDPE conversion is plotted as a function of temperature. The observed differences resulted from the properties inherent to zeolites, mainly textural and acidic features. The comparison of the temperature with 50% conversion (T_50%_) for all the zeolites β studied and pure LDPE shows clearly the advantage in the use of the zeolite catalysts. The cracking temperature progressively shifted to lower values in the order β > β_0_ > β_G_ > β_G/HNO3._ Previous literature reports focused on the influence of microporous features such as the internal cavity volume and the dimensionality of the pore structure on the product selectivity in cracking reactions [30]. Propane cracking was found to be facilitated on acid sites located in the eight-ring channels over the 12-ring ones of H-mordenite. This was related to partial confinement of the transition states within smaller pockets [31]. In contrast, protons located in less confined voids of ZSM-5, e.g., in the intersection of the channels, contributed to the increased cracking rate of *n*-butane because of their lesser confinement [25]. The obtained results show that the improved catalytic performance of zeolite β_G/HNO3_ originated from the highly microcrystalline structure free of extra-framework Al species. The improvement in microenvironment properties was not only beneficial in terms of meaningfully enhanced confined sorption; the strength of the protonic sites was also significantly increased (Table 2). The correlation between catalytic activity in polyethylene decomposition (T_50%_) and the features inherent to the microporous environment, i.e., the strength of sites expressed by ∆υ(_CO•••OH_), as well as the V_micro_ values (Figure 4B), clearly pointed to the benefits provided by microporous characteristics. The results discussed above are in line with previous reports [32] on the cracking of low-density (LDPE) and high-density polyethylene (HDPE). High external surface area is of secondary importance when considering 12-ring zeolites as reported for hierarchical zeolites β obtained through base-leaching [32]. If quenching the microporous characteristics, i.e., the number of voids inducing the confinement effect, the catalytic ability can be significantly reduced. Derouane et al. [33] pointed out that the interaction between the adsorbate molecule and the zeolite surface is the strongest when the radius of the molecule and the surface curvature are comparable. The enhanced interaction leads to increased concentration of reactants in the neighborhood of the acid sites. The 3D micropore structure of the zeolite and its effect on sorption equilibrium have an additional great impact on the reaction rate, especially when the sorption energetics are magnified by the surface curvature [33]. Jones et al. [34], on the basis of the ensemble-averaged transition state energy determined for MFI, BEA, mordenite, ferrierite, faujasite, andchabazite, concluded that all these structures stabilize transition states with electrostatic interactions to similar extents. Because of the deprotonation energy, values do not depend on the proton acceptor; they provide an acid strength scale that is independent of the reacting or adsorbing molecules involved. Consequently, the authors ascribed the differences in the catalytic performance to the diversity of zeolite void shapes and sizes that confine transition states and reactive intermediates to different extents. The same shape and size of the confining voids in the zeolites β studied give rise to the same adsorption adducts. Then, the differences in the Si(OH)Al hydroxyls strength should be considered as of the primary importance. Diffusional restrictions for the LDPE chains, almost identical due to the same textural parameters, affect the catalytic cracking to a lesser extent than the strength of the sites located in the internal voids. The catalytic reactivity of Brønsted acid sites confined in zeolites is determined not only by their intrinsic chemistry but also by the specific topologic features of the zeolite matrix incorporating them. The proton location should be considered as a factor influencing the diversity in their catalytic behavior [35]. Furthermore, their spatial proximity can also substantially affect the properties of confined catalytic sites; adjacent Brønsted acid sites significantly enable central cracking over the terminal because of higher van der Waals stabilization energies [36].

**Figure 5:**
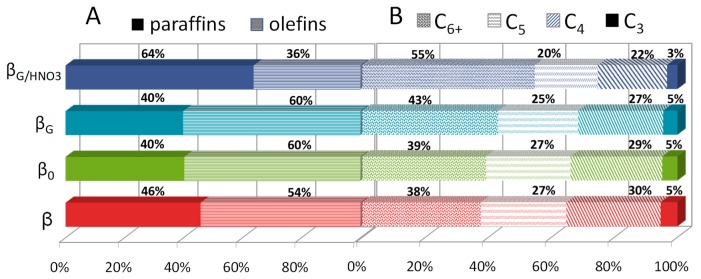
Distribution of paraffinic (solid symbols) and olefinic (pattern symbol) products (**A**) and the selectivity of the LDPE cracking over zeolites Beta studied (**B**).

The role of the strength of protonic sites can be visualized in Figure 4B where an additional decrease in conversion temperature was observed for the β_G/HNO3_ catalyst accommodating the protons of the strength increased by extra-framework material extraction while preserving the Al Lewis sites of high strength. The catalytic cracking reaction of LDPE on studied zeolites was followed in FTIR operando studies with GC–MS analysis of the resulting products (Figure 5). The FTIR/GC–MS operando studies were performed at 230 °C. For the LDPE cracking, the decisive factors were the density and the strength of acid sites of the catalysts, as polymer fragments were able to diffuse through all the system of channels. In other words, after preliminary cracking, the polymer fragments must diffuse through a micropore system to reach active sites with strong enough acidity for cracking these relatively refractory molecules. The speciation of acid sites governs the selectivity of the cracking process. The quantification of the reaction products by GC-MS showed that the catalyst β_G/HNO3_ predominantly yielded paraffin (Figure 5A). Lewis acid sites on the catalyst abstracted a hydride from the alkane and produced alkenes. The lower concentration of Lewis acid sites in β_G/HNO3_ reduced the dehydrogenation of alkanes, thereby providing a highly paraffinic fraction. The setting of Al atoms and their spatial proximity also affect the adsorption selectivity and, therefore, the selectivity of the overall catalytic process [36,37]. Adjacent Brønsted acid sites favor cracking over dehydrogenation, as well as central cracking over terminal cracking. However, the unique feature of zeolites βare a poor representation of the single Al atoms in favor of the close unpaired Al atom. In zeolite β, the concentration of Al pairs can vary between 40% and 65% of the Al atoms (for Si/Al < 18) [38]. Therefore, adjacent Al atoms, i.e., both Al atoms in the pairs and closed unpaired Al atoms in zeolite β, can synergistically enhance the adsorption of central C−C bonds and their cracking. The bimolecular hydrogen transfer reactions require adjacent acid sites to facilitate interaction between two adsorbed molecules. These reactions also result in decreased olefin production. The less extensive recracking rate in β_G/HNO3_ can be assigned to the facilitated diffusion of reagents due to purifying the zeolite channels from extra-framework species (EFAL). In other words, the presence of bulkier EFAL moieties in the zeolite micropores can benefit the geometrical changes in the zeolite voids and finally impact the more efficient stabilization of the reaction intermediates and/or transition states. This strongest interaction between the adsorbate and the zeolite surface can rule both the selectivity and the activity of the catalyst [39]. Furthermore, the enhanced interaction leads to increased concentration of reactants in the neighborhood of the acid sites. 

**Figure 6:**
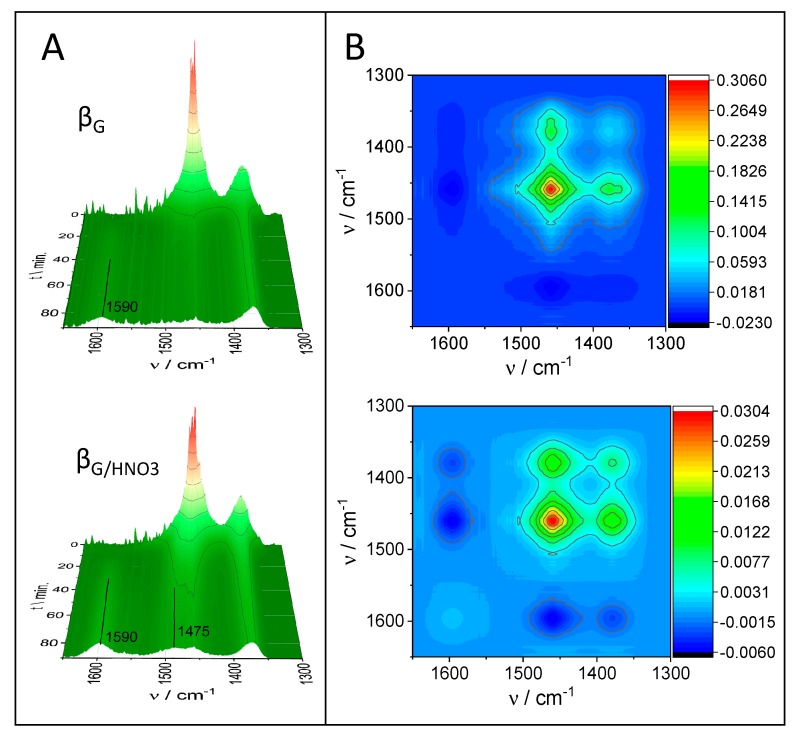
(**A**) Time-resolved FTIR spectra of LDPE cracking over zeolites β_G/HNO3_ and β_G_ during 80 min of the reaction. (**B**) The synchronous correlation two-dimensional (2D) spectra representing the coke residue formation in zeolites β_G/HNO3_ and β_G._

**Figure 7:**
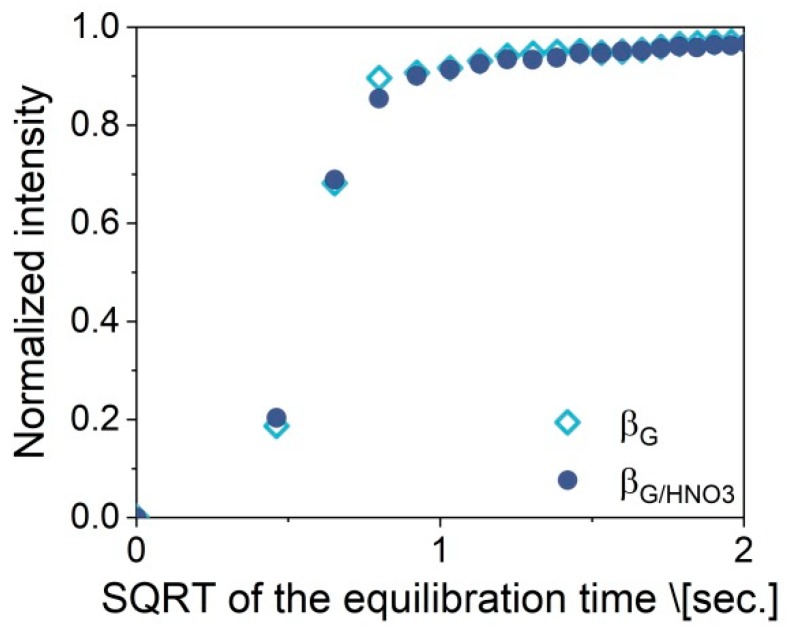
Dependence of the normalized intensity of 1450-cm^−1^ band of hexane on the square root of time for zeolites β_G/HNO3_ and β_G_. Hexane was sorbed at 100 °C.

Cracking of large polymer fragments on the external surface of the catalyst is associated with oligomerization, cyclization, and hydrogen transfer reactions. Carbonaceous deposits are generally known to induce catalytic deactivation of zeolites. The reaction followed by operando FTIR/GC–MS studies allowed for simultaneous monitoring of the catalytic performance and the build-up of carbonaceous deposits on the surface. The lower amount of extra-framework Lewis acid species resulted in increased coke deposit formation. The coke deposit evolution was followed by the 1590-cm^−1^ band assigned to of C=C band of aromatic compounds that cannot be desorbed [40] (Figure 6). Upon cracking of the LDPE, the disappearance of 1450- and 1375-cm^−1^ bands (CH_3_–) accompanied by the development of a 1590-cm^−1^ band on one-dimensional (1D) FTIR was observed (Figure 6A). The two-dimensional (2D) correlation analysis of FTIR spectroscopy results (Figure 6B) showed a negative cross-peak between these bands in the synchronous maps. The comparison of the 1D FTIR spectra of the β_G/HNO3_ and β_G_ spent catalysts within the 1300–1700-cm^−1^ region (Figure 6A) clearly shows that the new spectral features at 1475 cm^−1^ related to the alkyl aromatics [40,41] arose solely for zeolite β_G/HNO3._ The results show that the micropores of β_G/HNO3_ free of extra-framework species together with Brønsted sites of high strength enhanced bimolecular reactions, condensation, and cyclization, yielding more aromatic carbonaceous species. The diffusivity of the cracked linear LDPE chains should be reflected in their concentration changes as a time function inside the micropores. Therefore, hexane was used as a model compound and its diffusivity was measured by rapid scan FTIR spectroscopy by collecting one spectrum every 0.2 s. The sorption of hexane was followed using the 1450-cm^−1^ band intensity changes upon sorption at room temperature (RT) and 100 °C and 30 mbar of partial pressure. The values of the Q_t_/Q_e_ ratio (where Q_t_ represents uptake at time t and uptake after equilibration Q_e_) were plotted versus the square root of the time (Figure 7). In-line monitoring of hexane adsorption confirmed the same diffusivity of hexane in both β_G/HNO3_ and β_G_. With regard to the uptake of hexane, the model compound of linear products of the LDPE chain cracking was the same for both zeolites, as the textural features did not affect the hydrogen transfer and oligomerization reactions. Therefore, the yield of the aforementioned processes was ruled by the strength of Brønsted acid sites.

## 3. Materials and Methods

### 3.1. Materials

#### 3.1.1. Preparation of Beta Seeds

The zeolite βseeds were prepared from the mixture with a molar composition of SiO_2_: 0.02 Al_2_O_3_: 0.028 Na_2_O: 0.6 TEAOH: 0.2 HCl: 20 H_2_O. In a typical synthesis, 14.7 cm^3^ of 25% tetraethylammonium hydroxide (35% TEAOH, Sigma-Aldrich, Grajewo, Poland) was mixed with 2.25 cm^3^ of 3.70 M HCl (37%, POCh, Gliwice, Poland). Then, 2.5 g of fumed silica (Sigma-Aldrich, Grajewo, Poland) was added under vigorous stirring. After 30 min, 0.1906 g of sodium aluminate (NaAlO_2_, Riedel-de-Haën, Toruń, Poland) dissolved in 2.9 cm^3^ water was added, and the reaction mixture was stirred for the next 30 min. The obtained gel was aged at 150 °C for 22 h under static conditions, yielding the zeolite βprecursor.

#### 3.1.2. Functionalization: Zeolite β_G_

The seed solution was used in the functionalization of zeolite seeds by an organosilane. The produced seeds were functionalized with (3-glycidyloxypropyl)trimethoxysilane (GLYMO) (Innosil, Poznan, Poland), which was used in a molar percentage of 5% relative to the total amount of silicon in the seed gel. The functionalization of βseeds with the organosilane took place under reflux at 90 °C, for 6 h, with stirring.

The crystallization step was followed by hydrothermal treatment in fully Teflon-lined autoclaves at 150 °C for three days under static conditions. The obtained materials were filtered, washed with water, and dried in air at ambient temperature. The template of as-synthesized samples was removed by calcination at 550 °C (heating rate of 1 °C∙min^−1^) for 8 h in air.

The protozeolytic microporous βmaterial was synthesized according to the procedure described above without the modification step. The βseed mixture after cooling (without opening the autoclave) was crystallized in the same way as the modified samples. The sample after crystallization and calcination was denoted as β_0_.

Calcined samples were converted to their ammonium form by four-fold ammonium ion exchange with 0.5 M NH_4_NO_3_ at 60 °C for 1 h.

The commercial zeolite NH_4_Beta (Si/Al = 22, Zeolyst, Kansas City, KS, USA, CP814C) was denoted as β.

The most active catalyst β_G_ (250 mg) was leached with 0.05 M HNO_3_ solution (9 cm^3^) to extract extra-framework Al Lewis acid sites from the microporous environment.

Finally, the zeolites were filtrated, washed, dried at room temperature, and calcined at 550 °C for 5 h with a heating rate of 2 °C·min^−1^, in order to obtain the active proton form.

The low-density polyethylene (LDPE) was supplied by Alfa Aesar (Product No.: 42607, Lot No.: P28D047).

### 3.2. Methods

Elemental Si and Al concentrations in the materials were determined by the ICP-OES method with an Optima 2100DV (PerkinElmer, Krakow, Poland) spectrometer. The zeolite sample (80–100 mg) was digested with HCl (4 cm^3^, 35%) and HF (0.4 cm^3^, 48%). The final weight of the solution was adjusted with deionized distilled water. Wide-angle XRD patterns were obtained with a Rigaku Multiflex diffractometer equipped with Cu Kα radiation (40 kV, 40 mA). Nitrogen sorption measurements at −196 °C were performed on a Quantachrome Autosorb-1-MP gas sorption apparatus. Prior to the measurements, all samples were degassed under high vacuum (10^−5^ mbar) at 350 °C for 16 h. The micropore volume was calculated based on the *t*-plot method. The apparent specific surface area was determined according to Brunauer–Emmet–Teller (BET) method considering the recommendations of Rouquerol et al. [17]. The pore size distribution and pore volume (V_EXT_, range between 1.7 and 30 nm) were calculated via the Barrett–Joyner–Halenda (BJH) model using the adsorption branch [42]. Transmission electron microscopy (TEM) pictures were carried out using a Tecnai Osiris microscope (FEI) with an X-FEG Schottky field emitter operated at 200 kV. Scanning transmission electron microscopy (STEM) images were performed using a high-angle annular dark-field (HAADF) detector. The materials were dropped onto a holey carbon film supported on a copper grid (Agar Scientific, 300 mesh). Prior to FTIR studies, the materials were pressed into the form of self-supporting discs (ca. 10 mg/cm^2^) and pre-treated in situ in a custom-made quartz IR cell at 500 °C under vacuum conditions (10^−5^ mbar) for 1 h. The spectra were recorded with a resolution of 2 cm^−1^ with a Vertex 70 spectrometer (Bruker, Poznan Poland) equipped with an MCT detector. All the spectra presented in this work were normalized to 10 mg of sample. The sorption of CO (PRAXAIR, 9.5) was performed at −100 °C up to the total saturation of the Lewis acid sites, up to maximum intensities of the 2230–2220-cm^−1^ and 2190-cm^−1^ bands, and the appearance of the bands of CO bonded to Brønsted acid sites (2175 cm^−1^). The total concentrations of Brønsted and Lewis acid sites were determined from quantitative IR studies of pyridine (Py) sorption (≥99.8%, Sigma-Aldrich Grajewo, Poland) [43,44]. The quantitative procedure involves the Py-gas sorption at 170 °C in an amount sufficient to neutralize all acid, followed by the desorption at the same temperature to remove the gaseous and physisorbed Py molecules. The band intensities in the latter spectrum were used to calculate the total concentration of Brønsted and Lewis sites, using the intensities (band height) of the 1545-cm^−1^ band of pyridinium ions (PyH^+^) and the 1450-cm^−1^ band of Py coordinatively bonded to Lewis sites (PyL). The following absorption coefficients were applied: 0.07 cm^2^∙µmol^−1^ for the 1545-cm^−1^ band of pyridinium ion (PyH^+^) and 0.10 cm^2^∙µmol^−1^ for the 1450-cm^−1^ band of pyridine coordinatively bonded to Lewis sites (PyL). The low-density polyethylene (LDPE) decomposition was studied in an operando system connected to a flow set-up. The self-supporting wafers (ca. 5.5–6 mg/cm^2^) prepared by mixing of the zeolitic catalyst with LDPE (1:1) were placed in a custom-made IR quartz gas cell of 1-cm^3^ volume. This IR reactor cell enables the simultaneous analysis of the gas phase and the catalytic surface under operating conditions. The nitrogen used as the carrier gas (30 mL/min) was introduced into the 1/16 lines (heated at 110 °C). Time-resolved IR spectra were collected with a Vertex 70 Bruker FTIR spectrometer, equipped with MCT detector. The scanner velocity of 80 kHz allowed for the accumulation of one spectrum (100 scans) every 0.5 min. The spectral resolution was 2 cm^−1^. The experiments were carried out at atmospheric pressure. The analysis of the species formed during polyethylene decomposition was supported by IR gas cell (PIKE, Madison, WI, USA) and gas chromatography (Agilent, Warsaw, Poland). The analysis was carried out at 230 °C up to total LDPE decomposition. The 2D COS plots were obtained with the use of OPUS 3D software from Bruker Optics and OriginPro9.1.

The catalytic cracking of LDPE was evaluated by thermogravimetric analysis in a PerkinElmer Pyris 1 analyzer (PerkinElmer, Krakow, Poland). A stock mixture of the ground polymer and the zeolite powder was prepared in a ratio of polymer–zeolite = 3:1; this was prepared by intimately mixing in an Agatha mortar. A certain portion of the mixture (typically 5–10 mg) was loaded in a 70-µL α-Al_2_O_3_ crucible and weighted with a five-digit Mettler Toledo balance before the analysis. The sample was placed in the furnace, the analytic gas was switched on, and the temperature was raised from 30 to 600 °C at a heating rate of 5 °C/min under a nitrogen flow of 80 mL/min STP. The conversion was calculated by deducting the catalyst weight and moisture content. The coke content of spent catalysts was measured in TG experiments where the temperature was raised to 800 °C, with a rate of 10 °C/min, and in synthetic airflow (80 mL/min). The amount of coke for each sample did not exceed 3.7% and was included in the conversion values.

## 4. Conclusions

The accumulation of high-strength Si(OH)Al sites in zeolite β in a well-developed microporous environment benefited the reduction of maximum polymer decomposition temperature. The individual effects of textural parameters and acidity on the catalytic performance were decoupled to present the respective impacts. Through strategic comparison of purely microporous zeolites with tuned microporous characteristics, we showed that the catalytic cracking of LDPE is dominated by the acidic feature inherent to the microporous 12-ring pore system. The interplay between activity, selectivity, and stability in microporous zeolites β can be optimized by relatively simple bottom-up synthesis modifications. Well-developed microporous characteristics and micropores free of extra-framework material assure the presence of the Si(OH)Al groups of high strength. Among catalysts of the same structure, whereby the impact of hydrocarbon and carbocation adsorption energies can be neglected, the differences in acid strength are actually relevant for catalytic reactivity.

## Figures and Tables

**Table 1:** Chemical composition and textural properties derived from N_2_ physisorption of the zeolites studied.

material	Si/Al ^a^	S_BET_^b^	S_micro_^c^	S_EXT_^d^	V_micro_^a^	V_EXT_^e^
		(m^2^·g^−1^)			(cm^3^·g^−1^)	
β	22	567	523	44	0.18	0.06
β_0_	19	667	535	134	0.19	0.11
β_G_	20	690	537	153	0.20	0.15
β_G/HNO3_	22	740	586	154	0.22	0.18

^a^ Si/Al ratio obtained from chemical analysis (ICP); ^b^ calculated via BET method with the recommendations of Rouquerol et al. [20]; ^c^ calculated from t-plot; ^d^ calculated as the difference between BET and S_micro_; ^e^ calculated from BHJ model.

**Table 2:** The amounts of Al derived from ICP analysis, the concentration of Brønsted (C_B_) and Lewis (C_L_) acid sites, and the acid strength of the Si(OH)Al groups ∆υ(_CO•••OH_).

Material	Al^a^	C_B_	C_L_	C_B_ + C_L_	Py_450_/Py_170_	∆υ(_CO•••OH_)
	(µmol·g^−1^)	(µmol·g^−1^)				(cm^−1^)
β	675	395	220	615	0.80	309
β_0_	762	405	236	641	0.84	321
β_G_	756	398	230	628	0.86	324
β_G/HNO3_	670	390	124	534	0.90	330

## Supplementary Materials

The following are available online: Figure S1: TEM/STEM micrographs.
