# Supplementary material for: Evaluation of the Textural Parameters of Zeolite Beta in LDPE Catalytic Degradation: Thermogravimetric Analysis Coupled with FTIR Operando Studies
Source: Molecules. 2020 Feb 19;25(4):926. doi: 10.3390/molecules25040926 (PMC7070239; doi:10.3390/molecules25040926)
Supplement: Supplementary file 1 [file molecules-25-00926-s001.pdf]

Supplementary Information

Evaluation of the Textural Parameters of Zeolite Beta in LDPE Catalytic Degradation: Thermogravimetric Analysis Coupled with FT-IR Operando Studies

Kamila Pyra [1,*], Karolina A. Tarach [1], Ewa Janiszewska [2], Dorota Majda [1], Kinga Góra-Marek [1,*]

[1] Faculty of Chemistry, Jagiellonian University in Kraków, 31-007 Kraków, Poland;

[2] Faculty of Chemistry, Adam Mickiewicz University in Poznan, 61-712 Poznan, Poland

* Correspondence: kamila.pyra@doctoral.uj.edu.pl (K.P.); kinga.gora-marek@uj.edu.pl (K.G.-M.)

Received: 22 January 2020; Accepted: 15 February 2020; Published: 19 February 2020

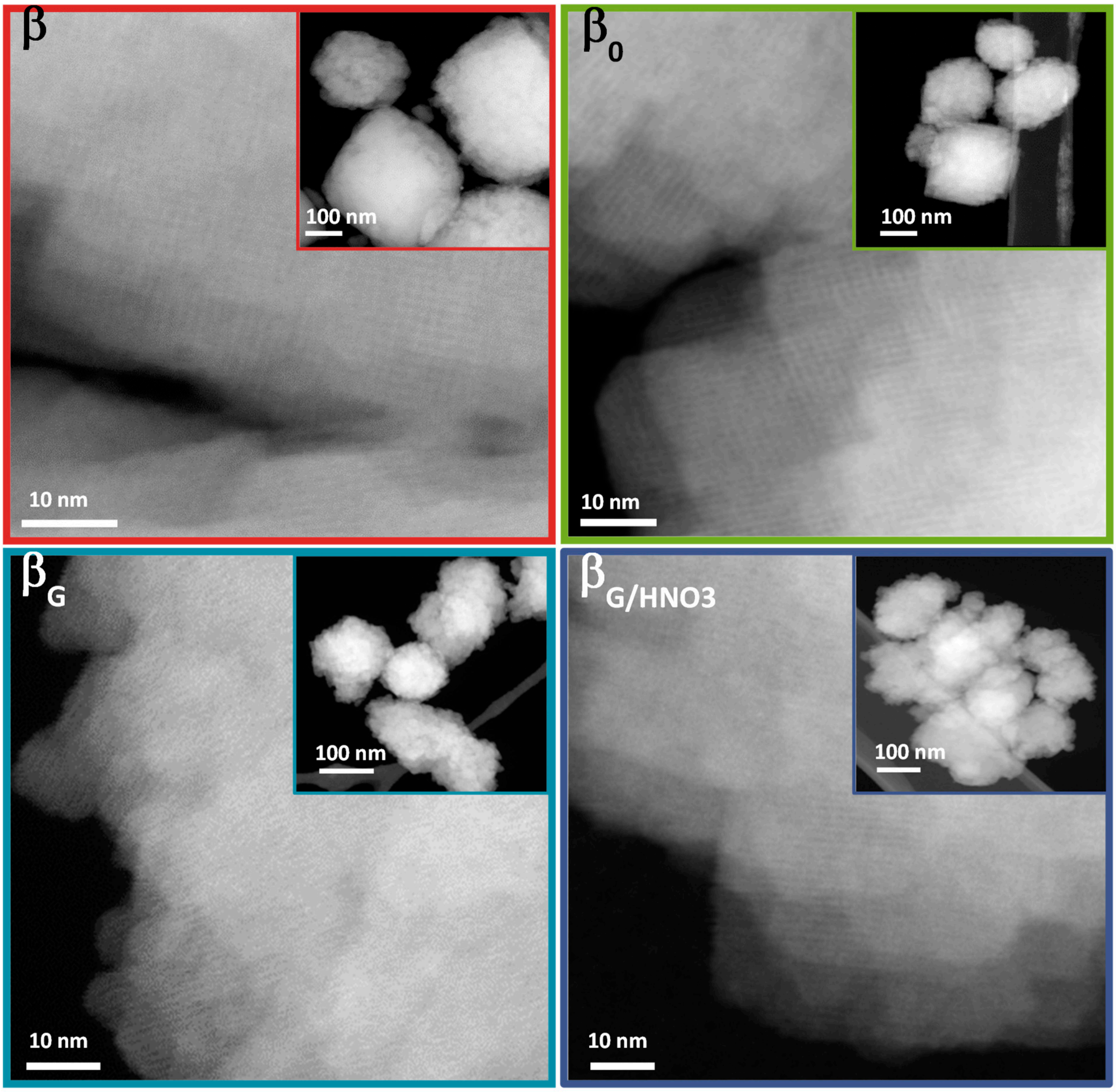

Figure S1. TEM/STEM micrographs.
